# The Role of Interleukin-6 Inhibitors in the Treatment of COVID-19 Infections: A Case Series

**DOI:** 10.7759/cureus.8631

**Published:** 2020-06-15

**Authors:** Satish Tadepalli, Jagan Mohan Rao Vanjarapu, Anna De Dona, Pramil Cheriyath, Vinod Nookala

**Affiliations:** 1 Internal Medicine, Hackensack Meridian Health - Ocean Medical Center, Brick, USA; 2 Internal Medicine, Community Medical Center, Toms River, USA

**Keywords:** covid 19, novel coronavirus, sars-cov-2, pneumonia, hypoxemic respiratory failure, tocilizumab, interleukin (il)-6, inflammation, pathophysiology

## Abstract

An outbreak of severe acute respiratory distress syndrome coronavirus 2 (SARS-CoV-2) infection started in December 2019 in China that resulted in a global health emergency. The World Health Organization later named the disease as coronavirus disease 2019 (COVID-19). Currently, there is no effective treatment available and the data are evolving through continuous clinical trials and ongoing research. Severe infections present with hypoxemic respiratory failure from acute respiratory distress syndrome as one of the major complications. We report two cases of COVID-19 patients who initially presented with moderate to severe symptoms. Later, their clinical course worsened despite ongoing treatment with multiple medications such as hydroxychloroquine and azithromycin until they were started on tocilizumab. Within a short period after they were administered tocilizumab, their oxygen saturation improved and other inflammatory markers such as D-dimer levels, lactate dehydrogenase, and ferritin levels decreased. There is an increase in the amount of research citing the role of various cytokines in the pathophysiology of COVID-19. Targeting the inflammatory mediators in the pathogenesis, especially interleukin-6 pathway inhibitors, would improve overall morbidity and mortality, thus decreasing the burden on healthcare systems.

## Introduction

Coronavirus disease 2019 (COVID-19) is caused by severe acute respiratory syndrome coronavirus 2 (SARS-CoV-2), which initially started as an outbreak of respiratory illness in Wuhan, China, and has rapidly spread globally, resulting in a pandemic. The optimal treatment for COVID-19 is still uncertain, and the data are evolving through continuous clinical trials and ongoing research. According to a report from a cohort of approximately 44,600 confirmed patients in China, the case fatality rate was around 2.3%, but this varies based on the demographics and underlying comorbidities [1]. Currently, each patient is treated on a case by case basis with medications such as hydroxychloroquine, azithromycin, and antiviral drugs, or in some cases with convalescent plasma therapy [2]. The novel coronavirus is believed to cause a cytokine storm, thus triggering an exaggerated immune response in the host [3]. Severe COVID-19 patients present with hypoxemic respiratory failure from acute respiratory distress syndrome as one of the major complications and other issues, such as acute kidney injury, liver failure, and cardiac injury. In a single-center study done by Luo et al, tocilizumab, a monoclonal antibody against interleukin-6 (IL-6) receptors, was shown to be effective, especially in people with severe illness [4]. Targeting these inflammatory mediators such as IL-6 will result in a decreased inflammatory response, thus minimizing the rate of respiratory complications, such as acute respiratory distress syndrome. This will improve overall clinical outcomes as well as decrease the burden on healthcare systems as it decreases the need for oxygen delivery/respiratory support systems. 

## Case presentation

Case 1

**Figure 1:**
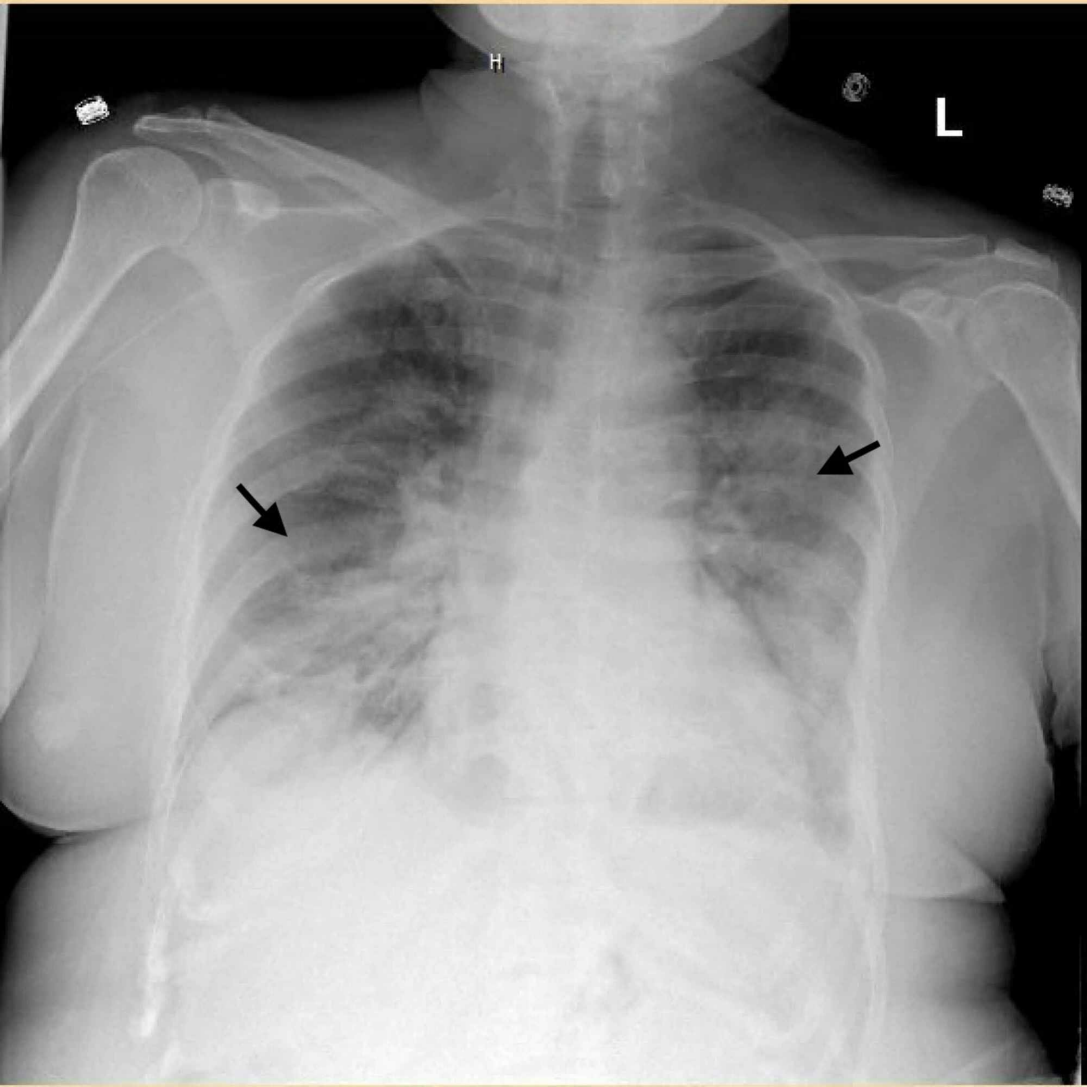
Portable chest X-ray of the patient Chest x-ray showing bilateral airspace disease with more prominence in the right basal and left central and basal regions.

A 62-year-old female presented to the emergency department with complaints of nausea, vomiting, diarrhea, and fever for five days. The symptoms started gradually and steadily got worse over a period of five days. A week before the admission, her husband tested positive for SARS-CoV-2. Her past medical history was significant for atrial fibrillation on apixaban, antiphospholipid syndrome, breast cancer status post lumpectomy and radiation, diverticulitis, hypertension, and rheumatoid arthritis. Upon admission, her vitals showed a temperature of 97.9 degrees Fahrenheit, a pulse of 82 beats per minute, a blood pressure of 142/64 mm Hg, a respiratory rate of 18 breaths per minute, and an oxygen saturation of 93% on room air. Laboratory investigations showed a white blood cell count of 6.8 K/µL, neutrophils 77.8% with an absolute neutrophil count of 5.3 K/µL, and lymphopenia with lymphocytes 11.1% and an absolute lymphocyte count of 0.8 K/µL. Her liver function tests revealed mild elevation of alkaline phosphatase 69 U/L. Her chest X-ray on admission revealed patchy infiltrates with more involvement in the right basal and left central and basal regions (Figure 1). Her other lab investigations revealed a D-dimer level of 725 ng/mL, ferritin 675.7 ng/mL, and lactate dehydrogenase 372 U/L. Her nasopharyngeal swab test for SARS-CoV-2 by reverse transcriptase-polymerase chain reaction was positive. 

On admission, she was placed in isolation. She was started on azithromycin monotherapy. She was not given hydroxychloroquine as she was allergic to the drug. The patient continued to spike fevers every day since admission and her oxygen saturation ranged from 90% to 96% on room air. Her shortness of breath was getting worse gradually, and her oxygen demand increased from two liters on a nasal cannula to five liters on a nasal cannula on the fifth day. She was placed on a bilevel positive airway pressure (BiPAP) machine and transferred to a negative pressure room. At this point, she was given one dose of 400 mg of tocilizumab IV. Within the next 48 hours, her fevers trended down, and her symptoms started subsiding. Her other inflammatory markers such as D-dimer, ferritin, and C-reactive protein started trending down. On day 12, her shortness of breath improved and had a mild cough. On day 24, she was discharged home for self-isolation for the next two weeks with home oxygen therapy.

Case 2

**Figure 2:**
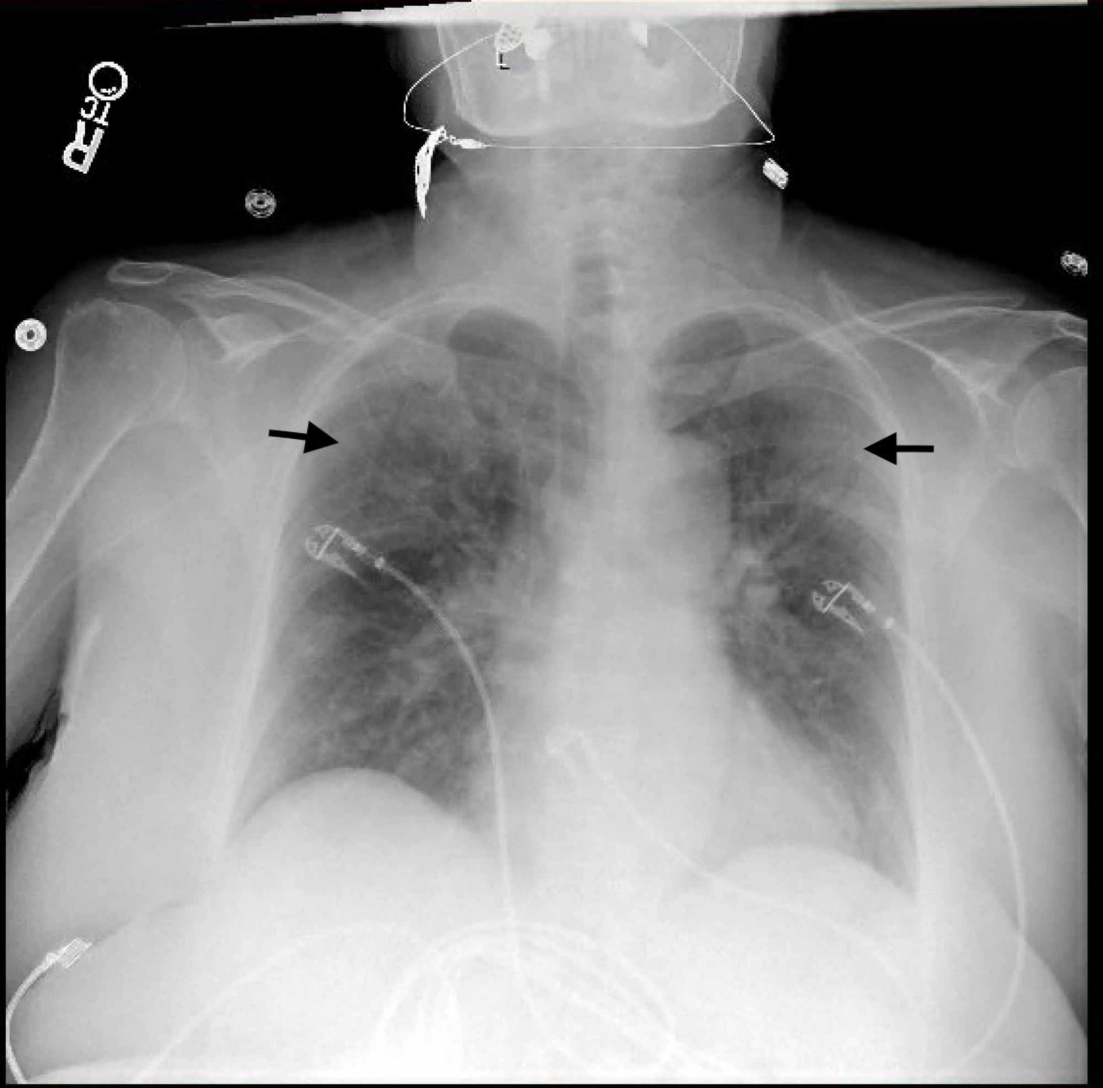
Portable chest X-ray of the patient Chest X-ray showing bilateral airspace disease.

A 65-year-old female was admitted from a rehabilitation facility with complaints of fever, cough, and shortness of breath after she acquired COVID-19 infection while getting physical therapy for fractures. Her symptoms started a week before she presented to the hospital and progressed gradually. Her past medical history was significant for hypertension, diabetes mellitus type II, asthma, and atrial fibrillation on apixaban. Upon admission, her vitals showed a temperature of 99.6 degrees Fahrenheit, a pulse of 68 beats per minute, a blood pressure of 116/73 mmHg, a respiratory rate of 17 breaths/minute, and an oxygen saturation of 100% on room air. Her laboratory investigations showed a white blood cell count of 4.1 K/µL, neutrophils 55.5% with an absolute neutrophil count of 2.3 K/µL, and lymphocytes 29.6% with an absolute lymphocyte count of 1.2 K/µL. Her liver function tests revealed elevated alkaline phosphatase 284 U/L. Her chest x-ray on admission showed bilateral airspace disease (Figure 2). Her other lab investigations revealed a D-dimer level of 3,514 ng/mL, ferritin 321.9 ng/mL, and lactate dehydrogenase 185 U/L. Her nasopharyngeal swab test for SARS-CoV-2 by reverse transcriptase-polymerase chain reaction was positive. 

On admission, she was started on hydroxychloroquine and azithromycin with vitamin C and zinc. Despite the treatment with multiple medications during her hospitalization, she started spiking fevers with a Temperature_max_ of 102 degrees Fahrenheit and started feeling tired. On the fourth day, she started feeling chest tightness and her oxygen saturation dropped to low 80s. A repeat chest x-ray showed bilateral airspace disease and her oxygen requirement increased from room air to two liters on the nasal cannula. At this point, she was given one dose of 400 mg of tocilizumab IV. Within the next 24 hours, her fevers started trending down, her oxygen saturation improved to mid-90s, and other inflammatory markers such as D-dimer, ferritin, and C-reactive protein started trending down. On day 12, she was discharged to the rehabilitation facility for further management and physical therapy.

## Discussion

The pathogenesis of SARS-CoV-2 infection is not well established, and a better understanding of this disease process will help in developing effective therapeutic options. The virus and the host factors are known to affect viral entry and pathogenesis. The SARS-CoV-2 virus binds to angiotensin-converting enzyme 2 receptors in the lower respiratory tract [5]. These viral particles initially invade the respiratory lining, and later involve other cells, causing a cytokine storm [6]. This results in a cascade of immune responses causing damage to various organs in the body, such as lungs, heart, and kidney, as well as respiratory failure and multi-organ failure. These are also associated with extensive tissue damage in alveoli [7].

There is an increasing amount of evidence showing its association with substantially elevated levels of various chemokines such as IL-1b, IL-6, IL-12, and IFN-g in serum, and its relation to lung injury in COVID-19 patients [8,9]. In a recent clinical study that was conducted in China, the plasma levels of COVID-19 patients showed marked elevation of IL-6, serum ferritin, and C-reactive protein in a major proportion of patients. In a retrospective study by Zhou et al., IL-6 levels were higher among non-survivors as compared to that of survivors [10]. Targeting these inflammatory mediators such as IL-6 will result in a decreased inflammatory response, thus minimizing the rate of respiratory complications such as acute respiratory distress syndrome [11].

Various drugs that target the chemokines involved in the disease process are under trials for the management of severe COVID-19 patients. Tocilizumab is a recombinant monoclonal antibody that binds to IL-6 receptors. This is currently being used in the management of various inflammatory conditions, such as rheumatoid arthritis, polyarticular juvenile idiopathic arthritis, polyarticular juvenile arthritis, and systemic juvenile idiopathic arthritis [12-14]. The proinflammatory cytokine IL-6 exerts its effects through IL-6 receptors. It plays an important role in the host defense, and its dysregulation has been implicated in the pathophysiology of various inflammatory conditions [11-14]. By interacting with IL-6 receptors, tocilizumab inhibits the binding of IL-6 to its receptors [15].

We report a case series of two patients with moderate to severe COVID-19 features that were successfully treated with tocilizumab. Initially, their clinical condition did not get better with other medications. The clinical condition of these patients started deteriorating with impending respiratory failure. When these patients were started on tocilizumab, their vitals started gradually improving with decreased oxygen support requirements. The usage of IL-6 inhibitors shows a promising therapeutic option, especially in patients with serious illness with impending respiratory failure. This will decrease the overall burden on healthcare systems.

## Conclusions

COVID-19 is a hyperinflammatory condition that usually affects the lungs. Targeting the inflammatory mediators involved in the pathogenesis, especially IL-6 pathway inhibitors, would help improve overall morbidity and mortality in these patients. We started treating our patients with tocilizumab considering various clinical indicators such as worsening of oxygen saturation (mid-80s), increase in oxygen requirement (>5 liters per minute on a nasal cannula), worsening chest X-ray or CT scan findings, and the rise of inflammatory markers, such as D-dimer, ferritin, and C-reactive proteins. More studies need to be conducted in order to stratify patients based on tocilizumab requirement and look for drug interactions with other medications.
